# Longitudinal change of inhibitory control functional connectivity associated with the development of heavy alcohol drinking

**DOI:** 10.3389/fpsyg.2023.1069990

**Published:** 2023-02-03

**Authors:** Luis F. Antón-Toro, Danylyna Shpakivska-Bilan, Alberto Del Cerro-León, Ricardo Bruña, Marcos Uceta, Luis M. García-Moreno, Fernando Maestú

**Affiliations:** ^1^Center for Cognitive and Computational Neuroscience (C3N), Complutense University of Madrid (UCM), Madrid, Spain; ^2^Department of Psychology, University Camilo José Cela (UCJC), Madrid, Spain; ^3^Department of Experimental Psychology, Faculty of Psychology, Complutense University of Madrid (UCM), Madrid, Spain; ^4^Department of Radiology, Faculty of Medicine, Complutense University of Madrid (UCM), Madrid, Spain; ^5^Department of Cellular Biology, Faculty of Biology, Complutense University of Madrid (UCM), Madrid, Spain; ^6^Department of Psychobiology and Methodology in Behavioral Science, Faculty of Psychology, Complutense University of Madrid (UCM), Madrid, Spain

**Keywords:** binge drinking, functional connectivity, MEG, inhibitory control, adolescence, heavy drinking

## Abstract

**Introduction:**

Heavy drinking (HD) prevalent pattern of alcohol consumption among adolescents, particularly concerning because of their critical vulnerability to the neurotoxic effects of ethanol. Adolescent neurodevelopment is characterized by critical neurobiological changes of the prefrontal, temporal and parietal regions, important for the development of executive control processes, such as inhibitory control (IC). In the present Magnetoencephalography (MEG) study, we aimed to describe the relationship between electrophysiological Functional Connectivity (FC) during an IC task and HD development, as well as its impact on functional neuromaturation.

**Methods:**

We performed a two-year longitudinal protocol with two stages. In the first stage, before the onset of HD, we recorded brain electrophysiological activity from a sample of 67 adolescents (mean age = 14.6 ± 0.7) during an IC task. Alcohol consumption was measured using the AUDIT test and a semi-structured interview. Two years later, in the second stage, 32 of the 67 participants (mean age 16.7 ± 0.7) completed a similar protocol. As for the analysis in the first stage, the source-space FC matrix was calculated, and then, using a cluster-based permutation test (CBPT) based on Spearman’s correlation, we calculated the correlation between the FC of each cortical source and the number of standard alcohol units consumed two years later. For the analysis of longitudinal change, we followed a similar approach. We calculated the symmetrized percentage change (SPC) between FC at both stages and performed a CBPT analysis, analyzing the correlation between FC change and the level of alcohol consumed in a regular session.

**Results:**

The results revealed an association between higher beta-band FC in the prefrontal and temporal regions and higher consumption years later. Longitudinal results showed that greater future alcohol consumption was associated with an exacerbated reduction in the FC of the same areas.

**Discussion:**

These results underline the existence of several brain functional differences prior to alcohol misuse and their impact on functional neuromaturation.

## Introduction

Heavy Drinking (HD) is a prevalent pattern of alcohol consumption among adolescents, which has become a major social and health concern. In adolescent population, such pattern of HD behaviors is characterized by the so-called Binge Drinking (BD) pattern, which consist in the ingest of at least four standard alcohol units (SAUs-10 mg ethanol-) in women, or five in men, within 2 h, followed by short abstinence periods (Courtney and Polich, 2009). It drives the organism into an ethylic intoxication state, with harmful neurobiological and neuropsychological consequences (Squeglia et al., 2014; Carbia et al., 2018; Almeida-Antunes et al., 2021). During adolescent neurodevelopment, the nervous system is engaged in a series of critical neurobiological changes, involving important high-order brain regions, making teenagers especially vulnerable to the outcomes of HD consumption (Blakemore and Choudhury, 2006; Crews et al., 2007). The development of the prefrontal, temporal, and parietal regions is particularly prominent during this period, characterized by delayed and progressive cortical thinning, and increasing structural connectivity (Spear, 2013). At the functional level, the adolescent brain shows a progressive reduction of electrophysiological local activation during cognitive tasks (Segalowitz et al., 2010). Along with this neurobiological maturation comes the improvement of higher-order executive functions. Despite the multiple definitions of executive functions, they can be understood as cognitive skills oriented to the planning, control, and supervision of complex thoughts and behavior (Jurado and Rosselli, 2007). Among other executive processes, inhibitory control (IC) is crucial in the behavioral regulation and proposed as a core function affected by and involved in the development of HD consumption (López-Caneda et al., 2014).

Some studies have evidenced the electrophysiological consequences of HD regarding IC, featured by a greater activation of supplementary motor areas, the right inferior frontal cortex (López-Caneda et al., 2012; Suárez-Suárez et al., 2020), as well as increased frontoparietal synchronization during the inhibition of alcoholic stimuli (Blanco-Ramos et al., 2022). Overall, these studies show altered neurofunctional activity during IC processes, despite not detecting differences in behavioral performance (see Almeida-Antunes et al., 2021) for a systematic review of electrophysiological signatures associated with BD consumption).

Nevertheless, despite the valuable evidence provided by those studies, their cross-sectional nature has some limitations when drawing causal inferences. Over the last decade, there has been raising concern about the existence of potential factors which predispose some individuals to engage in hazardous behaviors such as HD. In this scenario, albeit scarce, some longitudinal studies have provided evidence regarding neurobiological and neurocognitive differences predating HD. Focusing on IC, neuropsychological prospective works have highlighted poorer IC as predictive of future heavy drinking episodes (Squeglia et al., 2014; Peeters et al., 2015). Regarding neuroimaging studies, fMRI works reported reduced BOLD signal during IC tasks in frontal, parietal, and temporal regions predating HD, joint to an increase of BOLD response after HD initiation (Wetherill et al., 2013). Concerning electrophysiological evidence, previous MEG study evidence increased synchronization between frontotemporal regions during successful inhibitory responses before HD initiation, being associated with worse dysexecutive and impulsive symptomatology (Antón-Toro et al., 2021). However, electrophysiological studies exploring this important question from a prospective design, and even more so, studies from the perspective of functional connectivity, are still lacking.

Electrophysiological techniques, like magnetoencephalography (MEG), provide a direct measure of neural oscillatory activity with an excellent temporal resolution and good spatial resolution. These measures are optimal to explore fast oscillatory dynamics as those associated with IC processes (Cohen, 2011). Functional connectivity (FC), defined as the statistical dependence between the activity of two or more brain regions (Friston, 1994), discloses important information regarding dynamic neural synchronization. This allows studying the integrity of neural networks associated with relevant cognitive processes, and neuropsychiatric conditions (Baillet, 2017). Among the different FC metrics, the phase locking value (PLV) (Bruña et al., 2018), based on phase coupling theories, has shown the highest consistency and robustness applied to electrophysiological data (Garcés et al., 2016).

The current work aims to explore the relationship between the electrophysiological FC signatures during an IC task and heavy alcohol consumption, before and after its initiation. For this purpose, we conducted a two-year longitudinal study over a cohort of initially alcohol naïve adolescents. We recorded their electrophysiological activity by means of MEG during an IC task (go/no-go). We analyzed the relationship between their IC functional connectivity and the intensity of future alcohol consumption. Two years later, we studied the longitudinal change in FC between pre-and post-alcohol initiation stages, exploring its relationship with the intensity of alcohol consumption. According to previous reports, we expect an increased FC of fast oscillatory bands in core IC regions associated with higher consumption rates years later. After HD initiation, we hypothesize a switch in that pattern, with a greater decrease in FC in those adolescents who show greater consumption rates.

## Materials and methods

### Participants

We recruited an initial sample formed of 611 adolescents from different secondary education schools in the community of Madrid. All subjects reported no previous alcohol intake, familiar history of alcohol misuse, and no psychiatric or neurological disorders. All participants fulfilled the *Alcohol Use Disorder Identification Test* (AUDIT), and those who reported previous alcohol use episodes were discarded from the experiment. From this initial sample, 67 right-handed adolescents accepted to participate in the neuroimaging study. In the first stage (“Stage pre”), all participants completed again the AUDIT test and a personal semi-structured interview, in order to ensure that they had no previous alcohol use. Brain electrophysiological activity of each participant was recorded by MEG during the performance of an inhibitory control task go/no-go. Two years later, in the second stage (“Stage post”), 32 participants fully completed the experimental protocol. All 32 participants (mean age “stage pre” 14.6 ± 0.7; mean age “stage post” 16.7 ± 0.7) went through a similar protocol; they fulfilled the AUDIT test and a semi-structured interview to measure their alcohol consumption habits. Also, they completed a MEG recording during the go/no-go task. Based on the information from the AUDIT and the interview, for each participant, we calculated the number of SAUs ingested in a regular consumption episode. We took into consideration the number of drinks within 2–3 h and the type of beverage. Tobacco and cannabis consumption was controlled by means of self-reported tests and personal interview. Only four participants showed tobacco consumption in very low doses (less than 5 cigarettes per day), and three of those four participants had ever consumed cannabis (between one and eight joints in total). All participants were asked not to consume alcohol or other psychoactive drugs in the 48 h prior to the MEG recording (96 h in the case of cannabis use). All participants and their parents or legal guardians signed informed consent for each stage of the study, following the guidelines in the declaration of Helsinki. The ethical committee of the Universidad Complutense de Madrid approved the study.

### MEG recordings and data processing

MEG data were acquired using a 306-channel Elekta Neuromag system located in the Center for Biomedical Technology (Madrid, Spain), using an online anti-alias filter between 0.1 and 330 Hz and a 1,000 Hz sampling rate. Environmental noise was reduced using an offline signal space separation method (Taulu and Simola, 2006), and subject movements were compensated using the same algorithm. The acquired data were segmented into event-related epochs, and artifacted epochs were discarded from subsequent analyses. Only successful inhibitory trials were considered for further analysis. Additionally, only those participants with a performance accuracy (correct inhibitions) higher than 60% were considered for the final sample.

### MRI recording

A structural MRI was obtained from each participant using a General Electric Optima MR450w 1.5 T machine. Imaging protocol consisted in 3D T1-weighted high-resolution images with the following parameters: TE = 4.2, TR = 11,2 and TI = 450 ms, Flip angle = 12°, FoV = 100, acquisition matrix = 256 × 256, and slice thickness = 1 mm.

### Inhibitory task

**Figure 1:**
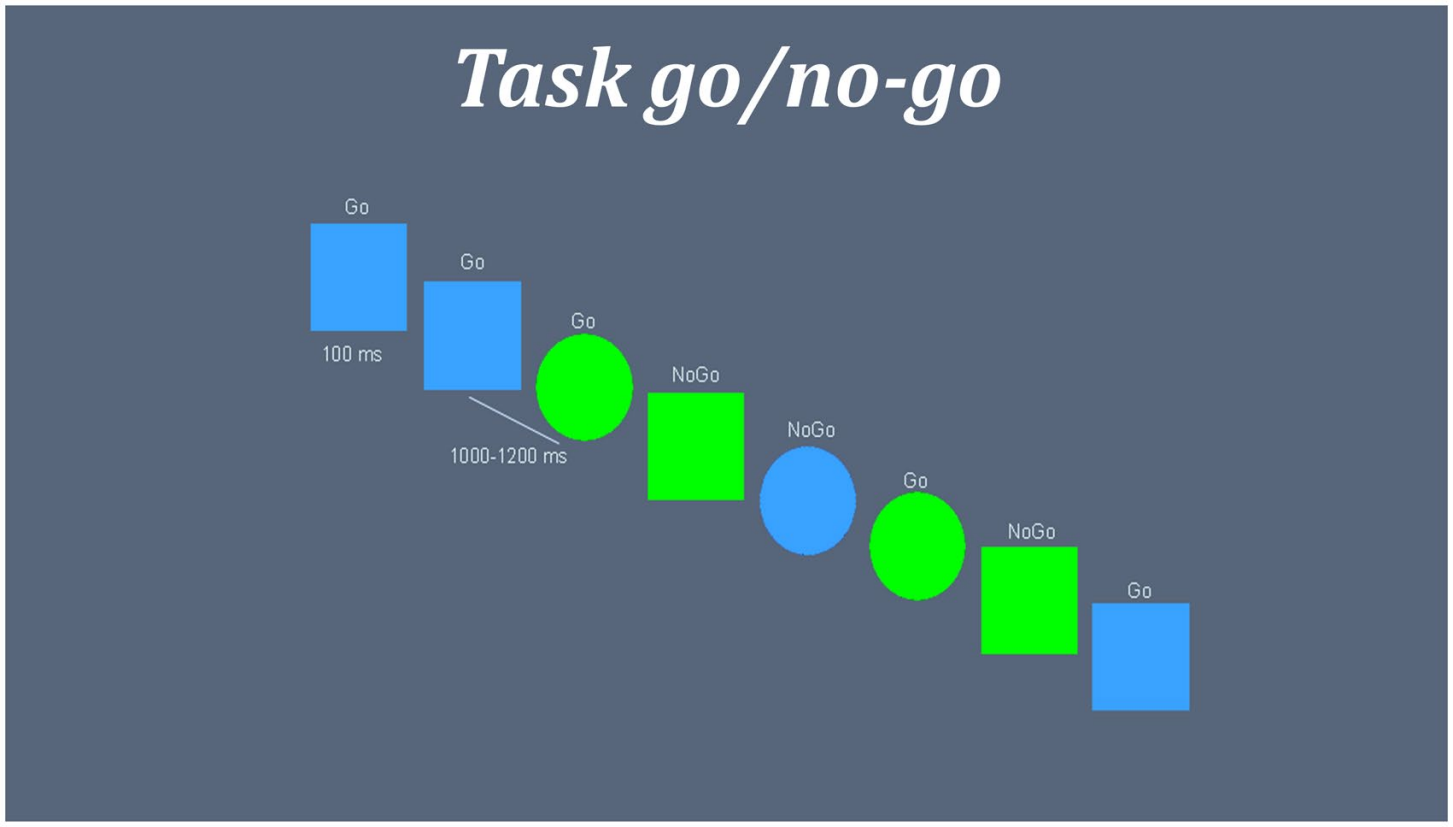
Representation inhibitory task go/no-go. Blue squares and green circles were set as “GO” targets, while green squares and blue circles were set as “NO-GO” targets. Inter stimuli interval were a random time between 1,000 and 1,200 ms. Stimulus were presented in screen during 100 ms followed by a fixation cross “+.” GO and NO-GO trials were presented randomly.

An equiprobable go/no-go task measured the performance of inhibitory networks in the subjects (Lavric et al., 2004; López-Caneda et al., 2012). A total of 225 “go” trials and 225 “no-go” trials were presented randomly in two different blocks. Figure 1 shows structure and time parameters of task presentation. All participants responded in the first block with their right hand. In the second block, they were commanded to respond with the left hand.

### Source-space reconstruction and functional connectivity analysis

We converted MEG signal into source-space using the subject’s native T1 and a single shell approach as forward model (Nolte, 2003). As inverse model, we used a linearly constrained, minimum variance (LCMV) beamformer in three classical bands: Alpha (8–12 Hz), Beta (12–30 Hz), and Low Gamma (30–45 Hz). The source model consisted of 1,204 cortical sources, labeled according to the automatic anatomical labeling (AAL) atlas (Tzourio-Mazoyer et al., 2002). Once calculated the spatial filter, we calculated the source-space functional connectivity matrix of successful inhibitory trials under the hypothesis of phase synchronization, using the PLV analysis (Lachaux et al., 1999; Bruña et al., 2018) in the time window of 250 to 350 ms after stimulus onset. We choose this time window based on previous literature, which highlights this temporal window as crucial in the inhibitory dynamics, and according to results of previous works (Antón-Toro et al., 2021), which evidence predominant differences in this time window. This metric is based on the study of the distribution of the instantaneous phase difference of two time-depending signals. If $\varphi_{1}\left(t,n\right)$ and $\varphi_{2}\left(t,n\right)$ are the instantaneous phase for instant t and trial n for signals 1 and 2, respectively, the value of PLV is given by:


$$PLV=\frac{1}{N}\sum_{n=1}^{N}\left|\frac{1}{T}\sum_{t=1}^{T}e^{i\left(\varphi_{1}\left(t,n\right)-\varphi_{2}\left(t,n\right)\right)}\right|$$

PLV value was calculated for each pair of cortical positions, obtaining a matrix of 1,204 × 1,204 for each frequency band and subject. From these matrices, we calculated the global FC for each cortical source, or “*nodal-strength,”* defined as the averaged FC of each cortical source with each other’s, obtaining a FC of 1×1,204 sources for each subject and frequency band. This analysis protocol was replicated in pre-and post-stages. In a second step, in order to assess the change of FC between “*Stage-pre”* and “*Stage-post*,” we calculated the symmetrized percent of change (SPC). This approach offers an index of the bidirectional rate of change between two-time points considering the time variance within the sample. An SPC value equal to zero means the absence of change in FC between stages, while positive values reflect an increase, and negative values a decrease. The higher the absolute values, the higher the change in that direction. This calculation resulted in an SPC matrix of 1,204 × 1,204 for each frequency band and subject, reflecting the corrected differences in terms of PLV between “*Stage-pre”* and “*Stage-post*.”

### Quality assessment for the source reconstruction

The beamformer is a spatial filter mapping the activity measured at the sensor level to the cortical space. Applying the beamformer spatial filter results on creating a single time series per source position, allowing to identify the brain activity related to a given cortical region. The correctness of this spatial filter is paramount in order to generate a trustworthy representation of the cortical activity, and with it a correct representation of the FC patters in brain space.

As the task used in this work was a visual go/no-go task, a related activity should appear in posterior regions of the brain, mainly calcarine fissure and occipital pole, approximately 100 ms after the presentation of the stimulus. In order to evaluate the quality of the spatial filter, we performed a source reconstruction of this response. For each participant, we reconstructed the source-space time series and calculated the average power of each source position between 80 and 120 ms after the presentation of the stimulus. This average value was normalized to the average power during the baseline, defined between 300 and 1 ms before the presentation of the stimulus. This gives us a value of activation for each source position.

**Figure 2:**
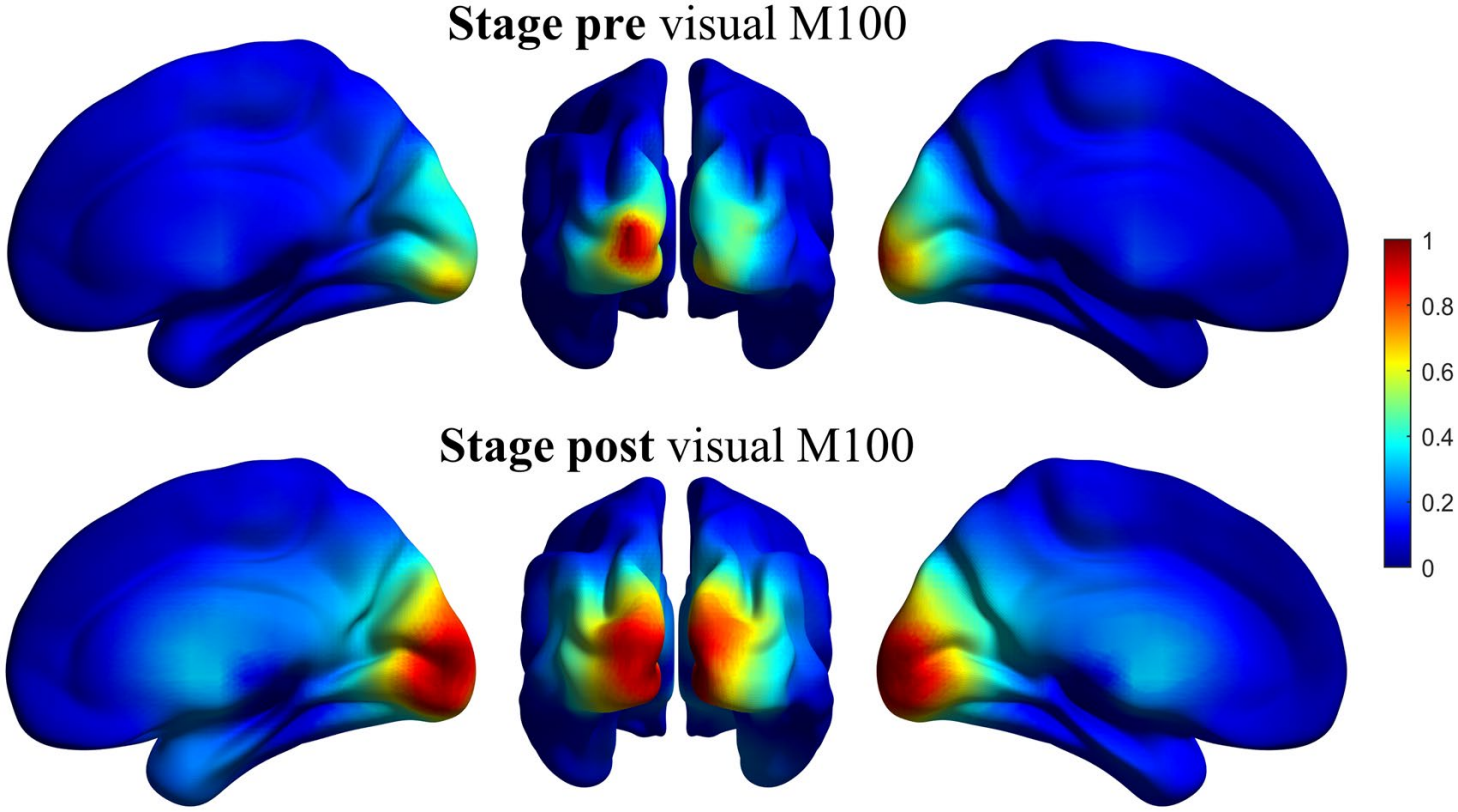
Representation of the source reconstruction of the primary visual response using the beamformer spatial filter for beta band. Grand average of the activity for all the participants. Left column: Ventral view of the right hemisphere. Middle column: Posterior view of both hemispheres. Right column: Ventral view of the left hemisphere. Superior line: pre-stage activity. Inferior line: post-stage activity. The activity is correctly reconstructed in occipital areas, mainly calcarine fissure. Similar results (not shown here) were obtained for the rest of the bands.

Figure 2 shows the result for this verification for the beta band in pre-and post-stages. The activity in the selected window maps correctly into visual areas, confirming the validity of the spatial filter. The rest of the bands are not displayed here but showed a similar behavior.

### Statistical analysis

Initially, we explored in out sample the relationship between demographic variables (sex and age) with alcohol consumption habits. We conducted a between-group *t*-test analysis using sex (male or female) as the independent variable (IV), and the number of SAUs as the dependent variable (DV). To study the influence of age on alcohol consumption, we performed three Spearman’s correlation analyses between age (pre-stage, post-stage, and age difference between pre-post stages) and the number of SAUs.

After, our main objective was to assess the relationship between the inhibitory control FC before alcohol use initiation, and the intensity of alcohol intake years later, as well as its relationship with the longitudinal changes of FC between both stages. First, using the “*Stage-pre”* FC matrix for each frequency band, we conducted a cluster based permutation test (CBPT) (Maris and Oostenveld, 2007) based on Spearman’s correlation analysis. This analysis allows identifying a cluster of cortical sources with significant correlations between FC and the level of future consumption in non-parametric datasets. Secondly, we used a similar approach using the SPC matrix for each frequency band. We conducted an unconstrained CBPT analysis using the whole SPC matrix (without cortical restrictions based on “*Stage-pre”* results). All results were corrected for multiple comparisons by a Bonferroni stepwise method. Only the clusters which survived this correction were reported as significant. PLV metrics are known to be affected by source leakage effects. This issue was controlled by the direct estimation of main source leakage confounders and by verifying its independence from our results. This analysis is shown in the Supplementary material; Supplementary Table S3.

Besides, in some analysis toolboxes (like Fieldtrip), permutation analyses such CBPT have some limitations controlling the interaction of multiple variables. The permutations of the data are not designed to maintain any data as belonging to the participant (covariables), so the result would be a random re-assignation of all the variables. For this reason, to study the association of FC measures and alcohol consumption with factors of sex, age, and task performance-related variables, we conducted two *post-hoc* analyses. First, we used a multivariate stepwise regression analysis, using the consumption ratio (number of SAUs) as the dependent variable. As predictors, we used FC and age in the pre-consumption stage, gender, as well as task performance variables, such as ratio of correct inhibitions (no-go trials) and responses (go trials), and response reaction time. Secondly, we applied a multivariate regression analysis using the change in FC between pre-and post-stages as the dependent variable. As predictors, we introduced sex, age pre-post change, and consumption rate.

## Results

### Demographic results

We tested the relationship between sex and age with the level of alcohol consumption. Analyses did not show significant differences between sexes and SAUs level. Age correlation analysis did not show significant results with SAUs level. Table 1 shows the demographic features and the results of these analyses.

**Table 1:** Demographic alcohol consumption results.

Stage	Sex		Age	
	*n* (*F*, *M*)	*T* (*p*)	Mean (sd)	rho (*s*)
“Stage-pre”	11 F, 22 M	−7.34 (0.469)	14.6 (0. 7)	−0.034 (0.855)
“Stage-post”	–	–	16.7 (0.7)	0.003 (0.986)
Stage pre-post	–	–	2.09 (0.29)	0.133 (0.467)

Between-groups *t*-test analysis using Sex as IV and SAUs as DV did not show significant differences. Correlations between SAUs with Age-pre, Age-post, or Age pre-post difference did not show significant results. SAU = Standard Alcohol Unit; IV = Independent variable; DV = Dependent variable; pre = pre-consumption stage; post = post-consumption stage.

### Functional connectivity results

We calculated global strength FC during an inhibitory control process both in the stage pre (before alcohol use initiation) and the stage post (2 years later, after alcohol use initiation). We analyzed the relationship of FC in the stage pre, and its change over 2 years between stages pre and post (SPC), with the level of alcohol intake in the “*Stage post”* (number of SAUs). Additionally, we studied the association of FC from each significant cluster with sex and age.

**Figure 3:**
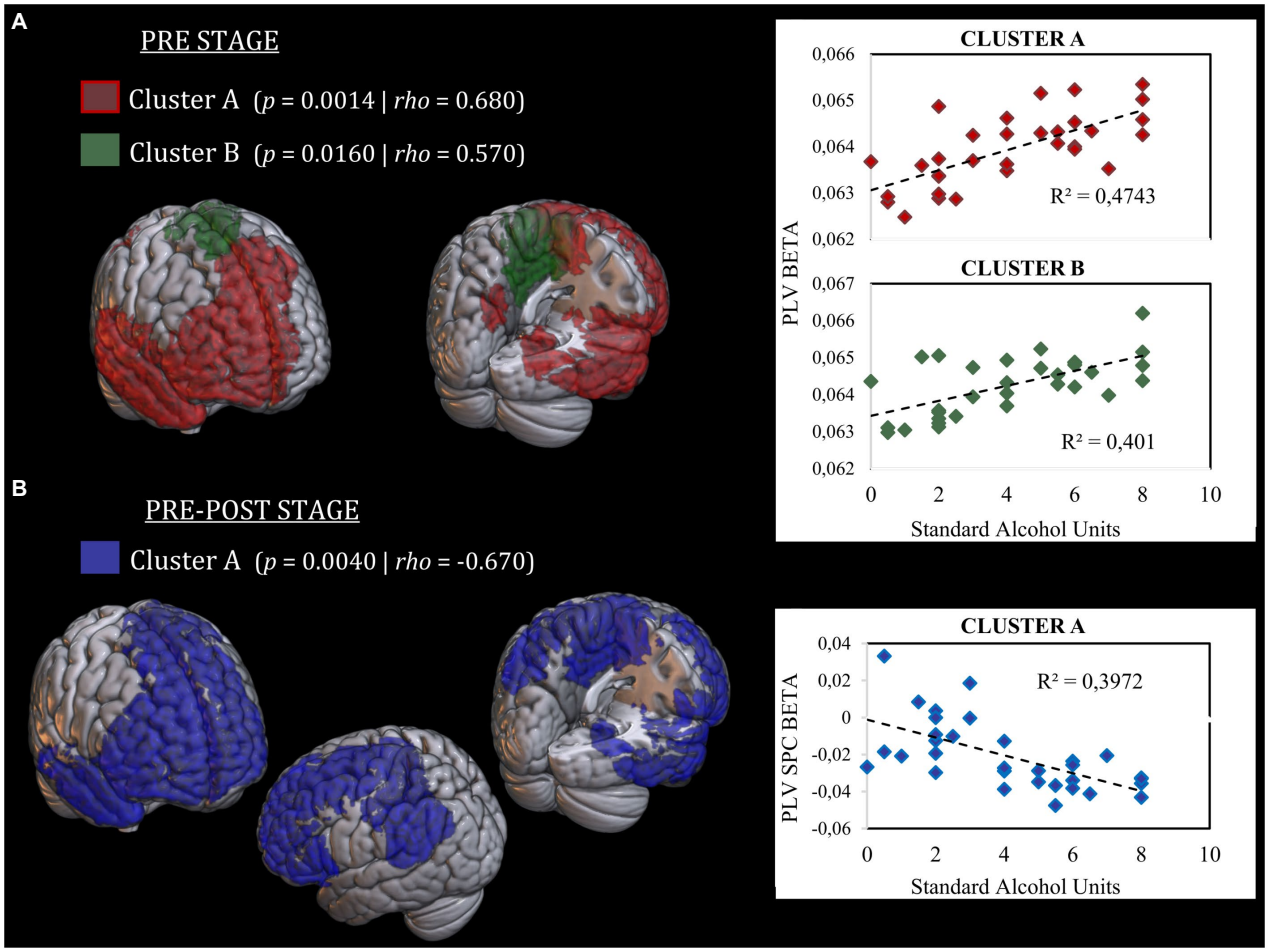
Representation of cortical distribution of significant clusters. **(A)** Significant clusters in the “*Stage-pre*” analysis. Cluster A is represented in red color. Cluster B is represented in green color. Both clusters show a positive correlation between FC and SAUs level. Scatter plots of correlation analysis are presented in the right side. **(B)** Significant clusters in the “*Stage pre-post*” analysis. Cluster A is represented in blue color. Cluster A show a negative correlation between SPC and SAUs level. Scatter plot of correlation analysis is presented in the right side. SAUs, Standard Alcohol Units; SPC, Symmetrized Percent of Change.

Regarding stage pre, the analysis revealed two clusters with positive correlations in the beta band with future alcohol use. The first cluster (cluster A) (*p* = 0.0014; *rho* = 0.680) was composed of 245 cortical sources mainly localized in the medial and right parts of the prefrontal cortex (superior, middle, inferior, and orbital frontal gyri), part of the left medial frontal gyrus, anterior and posterior cingulate cortices (ACC, PCC), right and medial temporal lobe (including hippocampus and parahippocampus), and parts of the right precuneus. The second cluster (cluster B) (*p* = 0.0160; *rho* = 0.569) was formed by 33 cortical sources located in the medial part of the somatosensorial and motor cortex, and the left middle cingulate cortex. Supplementary Table S1 depicts the percent of each region involved in each significant cluster. Both clusters indicate that the higher the FC in those regions, the higher the level of alcohol use 2 years later. Figure 3A shows the cortical distribution of each cluster and the graphical representation of each correlation.

Regarding stage pre-post, the analysis showed one cluster with a negative correlation in the beta band between connectivity SPC between both stages and the intensity of alcohol intake (*p* = 0.004; *rho* = −0.670). This cluster was composed of 290 cortical sources with a similar distribution to those found in both clusters of the stage pre. Additionally, this cluster encompassed a more expended part of the left prefrontal cortex (including the superior, middle, and inferior frontal gyri), the left inferior parietal gyrus, the left angular gyrus, and the left supramarginal gyrus. This cluster reflects that the higher the level of alcohol consumption, the greater decrease in FC between pre-and post-stages in those regions. Supplementary Table S1 depicts the percent of each region involved in each significant cluster. Figure 3B shows the cortical distribution of the cluster and the graphical representation of its correlation with alcohol use.

To ease the visual representation of the longitudinal relationship between FC and lower or higher alcohol intake, Supplementary Figure S1 represents the FC slopes between pre-and post-stages, subdividing the sample into low drinkers (SAUs <4) and heavy drinkers (SAUs > = 4).

### *Post-hoc* analysis results

Concerning *post-hoc* analyses, multivariate regression analysis of alcohol consumption predictors at pre-stage revealed a significant model where pre-consumption functional connectivity appears as a single and positive predictor of future alcohol consumption, explaining the 46% of the variance (*R*^2^_corr_ = 0.46). This means that higher FC at pre-stage would predicts heavier alcohol intake in the future. Table 2 shows results for this analysis. Regarding regression analysis of FC change predictors, results show two significant models. In the first one, consumption rate is the main predictor of the change in FC between pre-post stages, explaining the 32% of the variance (R^2^_corr =_ 0.32). Heavier drinking habits would be predictive of greater decrement of FC between pre-and post-stages. In the second significant model, consumption rate is the main predictor of FC change together with sex in a lesser degree, explaining the 42% of the variance (*R*^2^_cor =_ 0.42). Heavier drinking habits and being male would be predictive of greater decrement of FC between pre-and post-stages, Table 3 shows results of this analysis.

**Table 2:** Results of multivariate stepwise regression model at pre-stage.

Variables	*B*	E.T (*B*)	*β*	*t*	*p*	*R* ^2^_cor_
Constant	−138,429	27,204		−5.089	0.000	0.46
FC stage-pre	2,227,452	425,186	0.691	5.239	0.000**	
Inh_acc_pre			0.208	1.614	0.117	
Resp_acc_pre			0.038	0.287	0.776	
RT_pre			0.210	1.636	0.113	
Age_pre			0.101	0.730	0.471	
Sex			0.242	1.889	0.069	

Number of SAUs were used as DV. FC cluster at pre-stage, sex, age-pre, and task performance variables (inhibition and responses accuracy, and responses RT) were used as predictors. Higher FC at pre-stage were predictor of heavy drinking development years later. SAU = Standard Alcohol Unit; DV = Dependent variable; FC = functional connectivity; RT = reaction time; pre = pre-consumption stage; post = post-consumption stage.^**^p < 0.01.

**Table 3:** Results of multivariate regression model between prepost-stages.

Variables	*B*	E.T (*B*)	*β*	*t*	*p*	*R* ^2^_cor_
Constant	−0.004	0.007		0.531	0.559	0.42
SAUs	−0.005	0.001	−0.531	3.736	0.000**	
Age_diff			−0.144	−0.974	0.338	
Sex	−0.015	0.007	−0.313	−2.205	0.036*	

FC change (SPC) were used as DV. Number of SAUs, sex, and age pre-post difference were used as predictors. Higher alcohol intake was the main predictor of greater FC decrement between pre- and post-stages. Being male also predicts in a lesser degree greater FC decrement. SAU = Standard Alcohol Unit; DV = Dependent variable; FC = functional connectivity; RT = reaction time; pre = pre-consumption stage; post = post-consumption stage; SPC = Symmetrized percent of change.^**^p < 0.01, ^*^p < 0.05.

## Discussion

In this study, we aimed to describe the predisposing relationship between electrophysiological FC during inhibitory control (IC) task and the development of alcohol heavy drinking years later. Moreover, we analyzed the relationship between the longitudinal changes in FC and the intensity of alcohol misuse. In *Stage pre* (before alcohol use initiation), results showed that beta band FC in medial and right prefrontal regions and right temporal regions were positively associated with future alcohol consumption. This is, the higher the FC predating alcohol use initiation, the higher the intensity of future consumption. On the other hand, *Stage pre-post* analyses (FC changes between *stage pre*, and *stage post*, 2 years later) showed a negative relationship between changes in FC and the intensity of alcohol intake, in similar cortical regions (including left prefrontal and parietal cortices). Higher SAU levels were associated predominantly with negative and lower SPC values, pointing to a greater reduction of FC between pre and post stages. Conversely, lower SAU level was related predominantly to higher SPC values around zero, both positive and negative, indicating a more stable and lesser change in the FC between pre-and post-stages. Moreover, FC predating alcohol consumption and its changes along follow-up period were mainly associated with the posterior alcohol use intensity, being independent from the sex and the age of participants (both in pre-and post-stages).

Cognitive control, and especially IC, has been traditionally and predominantly associated with behavioral regulation abilities during adolescence. Several studies have linked the development of such cognitive skills with the anatomical and functional maturation of prefrontal, temporal, and parietal brain structures (Luna and Sweeney, 2004). Thus, deviances in the brain maturational course are associated with potential cognitive and behavioral dysregulation. In this vein, our results highlight the presence of an association between functional anomalies during the performance of IC processes and the development of risky behaviors, such as alcohol misuse. Similarly, these functional properties seem to show a distinctive evolution related to the intensity of alcohol consumption independently of sex or age. These results contribute to enriching the growing, albeit scarce, body of literature evidencing the existence of neurofunctional abnormalities in cognitive control processes associated with the development of HD behaviors.

At the time, there is a paucity of literature referring to electrophysiological and FC markers of predisposition to hazardous drinking in adolescents. To our knowledge, only one previous work explored this association, reporting higher beta band FC predominantly between right frontotemporal regions in those adolescents who transited into BD years later (Antón-Toro et al., 2021). In the current work, we adopted a non-group-based approach in order to explore the distribution of FC along the continuous variable of consumption, since the classification of healthy adolescents into binge drinkers may be to a certain extent artificially thresholded along this continuous variable. In line with this approach, current results seem to support likewise previous findings, highlighting a direct relationship between medial and right frontotemporal hyperconnectivity, and the development of future alcohol misuse. Interestingly, these cortical regions experience greater neuromaturation during adolescence and show important changes in their relationship with IC processes. In this vein, previous MEG studies have evidence of the maturational development of IC networks during adolescence. Vara et al. (2014) reported that adolescents show greater recruitment of right prefrontal and right temporal regions in the execution of go/no-go tasks compared with the adult population. Similar results were reported by Stevens et al. (2007) in an fMRI study, showing that adolescents, compared to adults, had a greater engagement of right ventrolateral and inferior prefrontal regions in inhibitory networks. This recruitment of core IC regions with supplemental and prolonged involvement of prefrontal and temporal areas in adolescents may be indicating an immature or deficient IC network.

Longitudinal results showed an exacerbated reduction of FC in previously hyperconnected areas as alcohol consumption becomes more intense. Compared with previous fMRI studies, current results evidence a similar pattern of brain functioning (although in opposite directions) before and after alcohol intake initiation. As reported by Wetherill et al. (2013) before BD onset, adolescents who transitioned into BD showed reduced BOLD activation during IC tasks in similar cortical regions, increasing in BOLD activity after a follow-up period of 3 years. These divergent results may be understood taking into account the different natures of BOLD and electrophysiological signals synchronization (slow fluctuations of hemodynamic flow vs. fast changes of oscillatory electromagnetic fields) and its complex relationship (Winterer et al., 2007). To our knowledge, there is no fMRI study that explored FC profiles during IC processes associated with HD development in adolescence, making it complicated to make solid inferences between both types of studies. Nevertheless, in spite of, and taking into account, above-mentioned differences between techniques, these studies converge underlining the existence of diverse brain functional abnormalities predating alcohol misuse, as the distinctive impact of alcohol consumption in adolescent neuromaturation.

Taking a broader perspective, outside the IC processes, as neuromaturation progress the FC properties of the adolescent brain go through significant changes. However, evidence provided by electrophysiological and hemodynamic studies seems sometimes contradictory, due mentioned reasons. In general, electrophysiological FC studies reports that functional networks tend to decrease or remain almost constant during adolescence (Machinskaya et al., 2019), while hemodynamical fMRI works evidence the opposite tendency, reflecting a progressive increase of intra-network FC (Stevens, 2016). Considering an electrophysiological perspective, the hyperconnectivity patterns found before HD seem to show that the development of consumption behaviors is associated with a lower cortical maturation of inhibitory networks. During the follow-up period, we found, in general, a decrease in the FC of the same cortical regions according to the expected, but very exacerbated in those adolescents who showed higher levels of alcohol use. These results seem to parallel with those reported by neuroanatomical studies. During adolescence, there is a normative loss of cortical and subcortical gray matter volume and cortical thickness, particularly within the prefrontal cortex (Giedd et al., 2010). In a longitudinal neuroanatomical study with adolescents, Pfefferbaum et al. (2018) found that heavy drinking initiation was associated with an exacerbated reduction of gray matter in the prefrontal, cingulate, and parietal regions. In this sense, after alcohol misuse initiation, there may be an alteration of the “normal” neurodevelopmental trajectories, characterized by overexuberant neuronal pruning. During adolescence, the synaptic pruning of excitatory contacts is a crucial event in the maturation of prefrontal circuitry. This process occurs through mechanisms of long-term potentiation (LTP), stimulating the formation of new connections, and long-term depression (LTD) which inhibits the functioning of excitatory synapses promoting its elimination (Lovinger and Abrahao, 2018). These plasticity processes are mainly dependent on glutamatergic and GABAergic neurotransmission, involving NMDA and GABAa receptors, which are particularly affected by ethanol pharmacodynamics (Lovinger and Abrahao, 2018). Several studies have demonstrated that the ingestion of low doses of ethanol (common in social drinking sessions) impairs the plasticity in multiple regions of the central nervous system (including dorsolateral PFC, motor cortex, etc.), enhancing canonical LTD and suppressing LTP (Lücke et al., 2014; Fuhl et al., 2015; Loheswaran et al., 2017). In this scenario, the recurrent ingestion of alcohol, even at low doses, would alter the normal neuromaturational course by impairing LTD and LTP mechanisms and subsequently increasing dramatically the “normal” rate of synaptic pruning. This may contribute to the sharp decline of FC observed in our results concerning the intensity of alcohol consumption. However, further experiments should explore in detail the relationship between the reduction of cortical thickness and FC regarding this matter. On the other hand, such a marked decline of functional synchronization in the prefrontal, temporal and parietal cortices may underlie some of the neurocognitive deficits associated with heavy alcohol consumption. In this sense, recent work has stated the importance of the ‘functional network stability’ during adolescent development, where longitudinally unstable or very changeable networks are predictive of worse cognitive performances and higher dimensional psychopathology (Fu et al., 2022).

Current work is not exempt from certain limitations. First, the reduced sample size, albeit large enough to depict distinctive FC patterns, should be increased in future research. Secondly, current works only provide evidence of functional synchronization. These results should be supported and complemented with further associations with neuropsychological and neuroanatomical data to have a wider perspective of the implications of such neurofunctional differences. Additionally, future studies should consider socioeconomic variables as potential predisposing factors and explore its interaction with neurofunctional profiles. Finally, we did not find statistical differences in the post-consumption stage. Given de sharp decline in FC, we could expect this trend to continue over time, evidencing more marked differences in heavy drinkers. Further studies should explore the evolution of FC in additional time points after HD initiation. This study also has several strengths. We use high temporal and spatial resolution techniques to explore the electrophysiological signatures of IC associated to HD development. This allows for depicting more precisely functional differences in very dynamic processes such as IC. Additionally, we used source-space analysis based on native anatomical data, providing a precise cortical localization of functional differences. This also would allow us in the future to explore the association of FC with neuroanatomical variables. Finally, the longitudinal approach of the current study enables making inferences regarding the potential neurobiological factors predisposing to HD and the divergent neuromaturational course associated with alcohol misuse.

In conclusion, heavy drinking is a concerning habit of alcohol misuse among adolescents, which causes, and consequences are complex and must be explored from multiple perspectives. In this work, we detailed electrophysiological connectivity anomalies associated with the development of BD and its impact on functional neurodevelopment. However, further research is necessary to fully understand the neurobiological cause of these differences.

## Data availability statement

The raw data supporting the conclusions of this article will be made available by the authors, without undue reservation.

## Ethics statement

The studies involving human participants were reviewed and approved by Universidad Complutense de Madrid. Written informed consent to participate in this study was provided by the participants’ legal guardian/next of kin.

## Author contributions

All authors of the present manuscript have made substantial contributions to its elaboration. LG-M and FM designed this research line and outlined main experimental stages, and approved the final version of this work. LA-T and LG-M recruited participants for this study and collected data and questionnaire evaluations. LA-T, RB, DS-B, AC-L, and MU performed MEG data pre-processing and analyses, while LA-T, DS-B, MU, and LG-M analyzed data from questionnaires. All authors contributed to the redaction and supervision of this manuscript.

## Funding

This research was funded by the Ministry of Health, under the framework of National Plan on Drugs (ref. PND2014|047 and PND2017|039).

## Conflict of interest

The authors declare that the research was conducted in the absence of any commercial or financial relationships that could be construed as a potential conflict of interest.

## Publisher’s note

All claims expressed in this article are solely those of the authors and do not necessarily represent those of their affiliated organizations, or those of the publisher, the editors and the reviewers. Any product that may be evaluated in this article, or claim that may be made by its manufacturer, is not guaranteed or endorsed by the publisher.
