# Supplementary material for: Longitudinal change of inhibitory control functional connectivity associated with the development of heavy alcohol drinking
Source: Front Psychol. 2023 Feb 3;14:1069990. doi: 10.3389/fpsyg.2023.1069990 (PMC9935580; doi:10.3389/fpsyg.2023.1069990)
Supplement: Supplementary file 1 [file Data_Sheet_1.PDF]

SUPPLEMENTARY MATERIALS

Distribution of significant clústers by AAL regions

Table S1A
'*Stage-pre*' Cluster A

Region AAL	nº sources	% of cluster	% of region
'Left Superior Frontal gyrus'	4	1,63	14,81
'Right Superior Frontal gyrus'	9	3,67	29,03
'Right Superior Frontal gyrus, Orbital'	3	1,22	100,00
'Left Middle Frontal gyrus'	5	2,04	14,71
'Right Middle Frontal gyrus'	14	5,71	35,00
'Right Middle Frontal gyrus, Orbital'	5	2,04	71,43
'Right Inferior Frontal gyrus, Triangular'	2	0,82	10,53
'Right Inferior Frontal gyrus, Orbital'	7	2,86	58,33
'Left Supplementary Motor area'	6	2,45	25,00
'Left Superior Frontal gyrus, Medial'	17	6,94	50,00
'Right Superior Frontal gyrus, Medial'	10	4,08	66,67
'Left Superior Frontal gyrus, Medial Orbital'	3	1,22	50,00
'Right Superior Frontal gyrus, Medial Orbital'	5	2,04	100,00
'Left Gyrus Rectus'	8	3,27	100,00
'Right Gyrus Rectus'	3	1,22	75,00
'Right Insula'	3	1,22	21,43
'Left Cingulate gyrus, Anterior part'	10	4,08	52,63
'Right Cingulate gyrus, Anterior part'	5	2,04	100,00
'Left Cingulate gyrus, Middle part'	2	0,82	12,50
'Right Cingulate gyrus, Middle part'	2	0,82	13,33
'Right Cingulate gyrus, Posterior part'	2	0,82	50,00
'Right Hippocampus'	7	2,86	100,00
'Right Parahippocampus'	10	4,08	100,00
'Right Calcarine fissure and surrounding cortex'	2	0,82	16,67
'Left Cuneus'	2	0,82	18,18
'Right Cuneus'	3	1,22	23,08
'Right Lingual gyrus'	5	2,04	27,78
'Right Superior Occipital lobe'	2	0,82	20,00
'Right Middle Occipital lobe'	3	1,22	17,65
'Right Fusiform gyrus'	14	5,71	73,68
'Right Superior Parietal gyrus'	3	1,22	16,67
'Right Angular gyrus'	1	0,41	5,56
'Right Precuneus'	8	3,27	38,10
'Right Heschl''s gyrus'	1	0,41	50,00
'Right Superior Temporal gyrus'	9	3,67	33,33
'Right Temporal pole, Superior Temporal gyrus'	8	3,27	100,00
'Right Middle temporal gyrus'	15	6,12	40,54
'Right Temporal pole, Middle temporal gyrus'	10	4,08	100,00
'Right Inferior Temporal gyrus'	17	6,94	65,38

Table S1A. Distribution of significant cluster A in the analysis of stage pre. *Nº sources* = number of cortical sources in the cluster corresponding to a specific region. *% Of cluster* = percent of the cluster corresponding to the nº of sources. *% Of region* = percent of the AAL regions corresponding to the nº of sources.

Table S1B
‘*Stage-pre*’ Cluster B

Region AAL	nº sources	% of cluster	% of region
'Left Supplementary Motor area'	5	15,15	20,83
'Right Supplementary Motor area'	2	6,06	14,29
'Left Cingulate gyrus, Middle part'	10	30,30	62,50
'Right Cingulate gyrus, Middle part'	3	9,09	20,00
'Left Cingulate gyrus, Posterior part'	1	3,03	20,00
'Left Postcentral gyrus'	1	3,03	2,94
'Left Precuneus'	1	3,03	3,57
'Left Paracentral lobule'	6	18,18	66,67
'Right Paracentral lobule'	4	12,12	50,00

Table S1B. Distribution of significant cluster A in the analysis of stage pre. *Nº sources* = number of cortical sources in the cluster corresponding to a specific region. *% Of cluster* = percent of the cluster corresponding to the nº of sources. *% Of region* = percent of the AAL regions corresponding to the nº of sources.

Table S1C
‘*Stage pre-post*’ Cluster A

Region AAL	nº sources	% of cluster	% of region
'Left Precentral gyrus'	1	0,34	3,13
'Left Superior Frontal gyrus'	10	3,45	37,04
'Right Superior Frontal gyrus'	10	3,45	32,26
'Left Superior Frontal gyrus, Orbital'	2	0,69	66,67
'Right Superior Frontal gyrus, Orbital'	3	1,03	100,00
'Left Middle Frontal gyrus'	12	4,14	35,29
'Right Middle Frontal gyrus'	16	5,52	40,00
'Left Middle Frontal gyrus, Orbital'	3	1,03	42,86
'Right Middle Frontal gyrus, Orbital'	6	2,07	85,71
'Left Inferior Frontal gyrus, Triangular'	1	0,34	4,76
'Right Inferior Frontal gyrus, Triangular'	6	2,07	31,58
'Left Inferior Frontal gyrus, Orbital'	4	1,38	33,33
'Right Inferior Frontal gyrus, Orbital'	8	2,76	66,67
'Left Supplementary Motor area'	10	3,45	41,67
'Right Supplementary Motor area'	2	0,69	14,29
'Left Superior Frontal gyrus, Medial'	16	5,52	47,06
'Right Superior Frontal gyrus, Medial'	11	3,79	73,33
'Left Superior Frontal gyrus, Medial Orbital'	4	1,38	66,67
'Right Superior Frontal gyrus, Medial Orbital'	4	1,38	80,00
'Left Gyrus Rectus'	7	2,41	87,50
'Right Gyrus Rectus'	4	1,38	100,00
'Left Insula'	1	0,34	7,14
'Right Insula'	4	1,38	28,57
'Left Cingulate gyrus, Anterior part'	12	4,14	63,16
'Right Cingulate gyrus, Anterior part'	4	1,38	80,00

'Left Cingulate gyrus, Middle part'	14	4,83	87,50
'Right Cingulate gyrus, Middle part'	8	2,76	53,33
'Right Hippocampus'	6	2,07	85,71
'Right Parahippocampus'	9	3,10	90,00
'Right Calcarine fissure and surrounding cortex'	1	0,34	8,33
'Right Cuneus'	2	0,69	15,38
'Right Lingual gyrus'	1	0,34	5,56
'Right Fusiform gyrus'	12	4,14	63,16
'Left Postcentral gyrus'	14	4,83	41,18
'Left Superior Parietal gyrus'	1	0,34	6,25
'Left Inferior Parietal gyrus'	13	4,48	72,22
'Left Supramarginal gyrus'	3	1,03	37,50
'Left Angular gyrus'	3	1,03	33,33
'Right Precuneus'	3	1,03	14,29
'Left Paracentral lobule'	5	1,72	55,56
'Right Paracentral lobule'	1	0,34	12,50
'Left Superior Temporal gyrus'	2	0,69	10,00
'Right Temporal pole, Superior Temporal gyrus'	6	2,07	75,00
'Left Middle temporal gyrus'	1	0,34	2,27
'Right Middle temporal gyrus'	4	1,38	10,81
'Right Temporal pole, Middle temporal gyrus'	10	3,45	100,00
'Right Inferior Temporal gyrus'	10	3,45	38,46

Table S1C. Distribution of significant cluster A in the analysis of stage pre-post. *Nº sources* = number of cortical sources in the cluster corresponding to a specific region. *% Of cluster* = percent of the cluster corresponding to the nº of sources. *% Of region* = percent of the AAL regions corresponding to the nº of sources.

Source leakage and volume conduction control

Phase synchronization metrics such PLV are known to suffer from source leakage effects, confounding the origin of the results. These biases used to be addressed by zero-lag-insensitive metrics, such as PLI (Stam et al., 2007) or ciPLV (Bruña et al., 2018). However, zero-lag-insensitive metrics have showed low test-retest reliability in MEG data (Colclough et al., 2016; Garcés et al., 2016), and might ignore bidirectional or indirect true connections (Gollo et al., 2014; Petkoski et al., 2018). For this reason, we employed a direct estimation of source leakage, calculating the spatial filters (beamformers) correlation and the activation power of our significant cluster (mayor confounders of source leakage effects). We used a multivariate regression model using FC of our significant cluster as dependent variable, and as predictors, spatial filters correlation, power, and alcohol use ratio (SAUs). FC was only predicted by alcohol use ratio, while beamformer correlation nor activation power did not show significant effect. Supplementary table 2 shows the results of this analysis.

Table S2.
Results of Multivariate stepwise regression model for source leakage

Variables	B	E.T (B)	*β*	t	*p*	R^2_{cor}
Constant	-.004	.007		.531	.559	.44
SAUs	,000	,000	,647	4,579	,000**	
Beamformer corr	,001	,001	,149	1,050	,303	
Power	,000	,001	-,041	-,300	,766	

Table S2. Control of source leakage effect. FC was used as dependent variable.

Figure S1.

Slopes of FC change

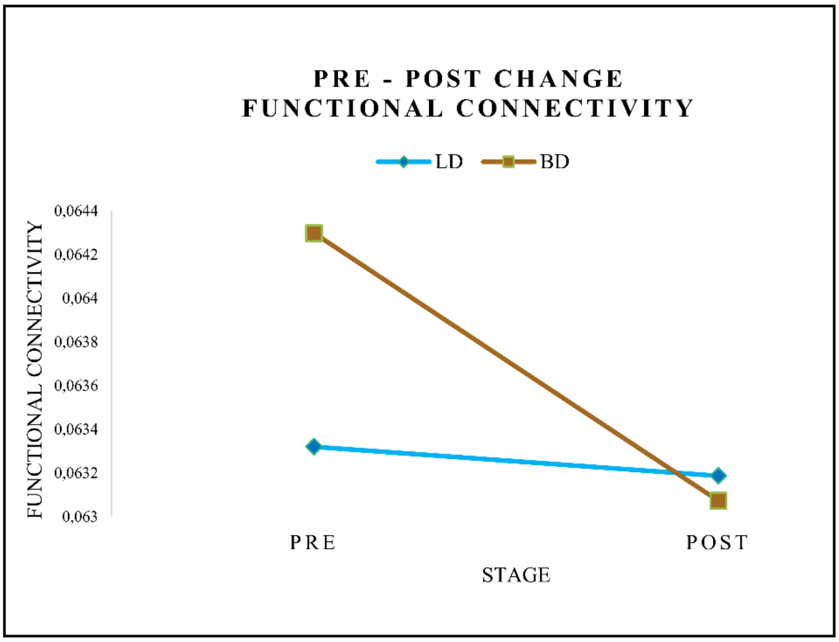

Figure S1. Representation of FC distribution between pre- and post-stages for light and binge drinkers. To represent slopes, sample were subdivided in two groups: Light drinkers (SAUs < 4), and Binge drinkers (SAUs >= 4). X axis represent the stage (pre or post alcohol use). Y axis represent FC values, extracted from '*Stage-pre*' significant clusters, and 'Stage-post' significant cluster. *SAU = Standard Alcohol Unit; FC = Functional connectivity.*

References

Bruña, R., Maestú, F., & Pereda, E. (2018). Phase locking value revisited: teaching new tricks to an old dog. *Journal of Neural Engineering*, *15*(5), 056011. https://doi.org/10.1088/1741-2552/aacfe4

Colclough, G. L., Woolrich, M. W., Tewarie, P. K., Brookes, M. J., Quinn, A. J., & Smith, S. M. (2016). How reliable are MEG resting-state connectivity metrics? *NeuroImage*, *138*, 284–293. https://doi.org/10.1016/j.neuroimage.2016.05.070

Garcés, P., Martín-Buro, M. C., & Maestú, F. (2016). Quantifying the Test-Retest Reliability of Magnetoencephalography Resting-State Functional Connectivity. *Brain Connectivity*, *6*(6), 448–460. https://doi.org/10.1089/brain.2015.0416

Gollo, L. L., Mirasso, C., Sporns, O., & Breakspear, M. (2014). Mechanisms of Zero-Lag Synchronization in Cortical Motifs. *PLoS Computational Biology*, *10*(4), e1003548. https://doi.org/10.1371/journal.pcbi.1003548

Stam, C. J., Nolte, G., & Daffertshofer, A. (2007). Phase lag index: Assessment of functional connectivity from multi channel EEG and MEG with diminished bias from common sources. *Human Brain Mapping*, *28*(11), 1178–1193. https://doi.org/10.1002/hbm.20346

Petkoski, S., Palva, J. M., & Jirsa, V. K. (2018). Phase-lags in large scale brain synchronization: Methodological considerations and in-silico analysis. *PLoS Computational Biology*, *14*(7), 1–30. https://doi.org/10.1371/journal.pcbi.1006160
